# Health-state utilities in a prisoner population: a cross-sectional survey

**DOI:** 10.1186/1477-7525-7-78

**Published:** 2009-08-28

**Authors:** Christopher AKY Chong, Sicong Li, Geoffrey C Nguyen, Andrew Sutton, Michael H Levy, Tony Butler, Murray D Krahn, Hla-Hla Thein

**Affiliations:** 1 Section of General Internal Medicine, Lakeridge Health Oshawa, Canada; 2 Faculty of Health Sciences, Queen's University, Ontario, Canada; 3 Faculty of Pharmacy, University of Toronto, Toronto, Ontario, Canada; 4 Mount Sinai Hospital Division of Gastroenterology, University of Toronto, Toronto, Ontario, Canada; 5 Johns Hopkins School of Medicine, Baltimore, Maryland, USA; 6 Ecology and Epidemiology Group, Department of Biological Sciences, University of Warwick, Warwick, UK; 7 Centre for Health Research in Criminal Justice, Sydney, Australia; 8 School of Public Health and Community Medicine, The University of New South Wales, Sydney, Australia; 9 Toronto Health Economics and Technology Assessment Collaborative, Department of Medicine Toronto, Ontario, Canada; 10 National Centre in HIV Epidemiology and Clinical Research, The University of New South Wales, Sydney, Australia

## Abstract

**Background:**

Health-state utilities for prisoners have not been described.

**Methods:**

We used data from a 1996 cross-sectional survey of Australian prisoners (n = 734). Respondent-level SF-36 data was transformed into utility scores by both the SF-6D and Nichol's method. Socio-demographic and clinical predictors of SF-6D utility were assessed in univariate analyses and a multivariate general linear model.

**Results:**

The overall mean SF-6D utility was 0.725 (SD 0.119). When subdivided by various medical conditions, prisoner SF-6D utilities ranged from 0.620 for angina to 0.764 for those with none/mild depressive symptoms. Utilities derived by the Nichol's method were higher than SF-6D scores, often by more than 0.1. In multivariate analysis, significant independent predictors of worse utility included female gender, increasing age, increasing number of comorbidities and more severe depressive symptoms.

**Conclusion:**

The utilities presented may prove useful for future economic and decision models evaluating prison-based health programs.

## Background

Prisoners represent an understudied population in health care research although they have a disproportionately high prevalence of many illnesses. For example, the prevalence of a wide-range of psychiatric disorders is easily more than double than that found in the community [1]. About 2% of the U.S. general population test positive for the hepatitis C antibody, compared to 12 to 64% of prisoners [2]. In particular, few investigations have explored the health-related quality of life (HRQL) of prisoners. Understanding inmate HRQL is essential to developing effective prison health programs and policies.

To the best of our knowledge, HRQL measurements in the form of utilities have not been obtained in prisoners. A utility is a preference-based, global measurement of overall health on a scale of 0 to 1, and is the most widely used method for evaluating HRQL in economic and decision analyses [3]. To date researchers have been limited to using utilities obtained from non-inmate populations [4,5]. However, the social, demographic, economic and health status of prisoners is clearly different from other groups [1,2]. Thus, specifically using inmate-based utilities could improve the validity and quality of economic and decision analyses that evaluate prison health programs.

The primary purpose of our study was to derive inmate-based utilities for use in future economic and decision models. Secondary objectives were to explore how socio-demographics and comorbidity affect prisoner HRQL and to compare different methods for deriving utilities.

## Methods

### Survey

Detailed methods of the survey have been published elsewhere [6]. Briefly, ethics approval was obtained from the Justice Health Human Ethics and Research Committee and the New South Wales (NSW) Department of Corrective Services Ethics Committee. In 1996, NSW Justice Health surveyed a cross-sectional random sample of prisoners from 27 male and two female correctional services, stratified for age, gender and indigenous origin. Participants were randomly chosen from a list of all inmates at each prison and those selected provided written consent; those that refused were replaced by inmates on a reserve list. Participants were compensated with $A 10.

Study nurses conducted extensive face-to-face structured interviews and participants completed various health questionnaires. Information collected included: 1) standard socio-demographic characteristics. 2) Comorbidities, gathered as self-reported health conditions. The survey also assessed whether prescribed medications for certain chronic health conditions had been used in the preceding two weeks, allowing us to further confirm some self-reported diagnoses. 3) Hepatitis C viral infection (HCV) status. The original purpose of this survey included assessing the prevalence of bloodborne infections, and HCV antibody and viral polymerase chain reaction (PCR) status were obtained through standard laboratory testing [6]. 4) Beck Depression Inventory (BDI). The BDI is a well-established 21-item questionnaire that assesses depression severity in the preceding week, with higher scores indicating more severe symptoms. The scores can then be divided into none, mild, moderate or severe symptom groups [7]. 5) World Health Organization Alcohol Use Disorder level, which classifies alcohol consumption in safe, harmful or hazardous categories [8]. 6) Short-Form 36 (SF-36). The SF-36 is a very widely used non-preference based general health survey that measures HRQL during the previous four weeks over eight domains [9].

Of the 789 patients in the original study, 55 did not complete the SF-36. Because the main purpose of this study was to derive utilities and the SF-36 was necessary to do so (see below), these 55 were excluded from the analysis for a final sample size of 734. The purpose of the original survey was to detect a range of health conditions from the NSW prisoner population and the sample size was thus chosen for that specific aim. The primary objective of this study is to provide an estimate of mean prisoner-based utilities; assuming an SF-6D standard deviation of 0.147 [10] and an acceptable error of 0.05, the needed sample size would be 33 [11], which is comparable to the usual recommendation of 30 to 60 subjects for standard gamble utility studies [12].

### Deriving utilities

Several methods exist for transforming SF-36 scores into utilities. These techniques attempt to translate the non-preference-based SF-36 health description into an accepted preference-based utility measurement. Existing techniques allow translating the SF-36 description into the Visual Analogue Scale, Health Utilities Index, Quality of Well Being Scores or Standard Gamble, all of which are distinct forms of measuring utilities. We selected what we felt were the two most robust methods, the Nichol method [13] and the Brazier SF-6D method [14]. The different techniques are not meant to be averaged together.

In the Nichol method, the eight SF-36 domain scores are first transformed into norm-based scores that have been standardized to the 1998 general United States (US) population for a mean of 50 and a standard deviation of 10. A regression equation developed from a sample of 6921 subjects is then applied to these eight scores to convert them into a Health Utilities Index II (HUI2) utility. The HUI2 is a multi-attribute health state classification system that defines 24 000 hypothetical unique health states and assigns a utility to each one using preference scores derived from a survey of the general population [15]. The utilities are based on the standard gamble, which is arguably the utility scaling method with the strongest theoretical foundation [3]. The Nichol translation of SF-36 scores predicts 50.5% of the variance in HUI2 utilities. The range of possible utilities using this method is -0.03 to 1.00.

Brazier's SF-6D method represents a more exact method of transforming SF-36 data into utilities. Respondent-level data from the SF-36 questions are first explicitly restructured into six health domains which describe 18 000 health states. Using the standard gamble, 611 members of the United Kingdom (UK) general population valued a 249 subset of these states, and a model was then developed to define utilities for the full set. Of existing methods for converting SF-36 data into utilities, this technique may be the most robust as it uses respondent-level data to clearly define unique health states which have been directly valued by a general population. Like the HUI2, the SF-6D represents community derived preferences for health outcomes. The range of possible utilities based on this model is 0.30 to 1.00.

### Analysis

To compare the distribution of categorical variables, contingency chi square analysis was used. When appropriate, t-tests or one-way analysis of variance with *post-hoc *Tukey tests were used to compare means of continuous variables. Pearson and Spearman tests were used to examine correlations between continuous variables. Statistical significance was defined at p < 0.05.

To assess predictors of utilities, all the socio-demographic and clinical characteristics were first assessed for significance in univariate analysis. All characteristics were then correlated with one another to assess for collinearity. Most clinical factors were found to be collinear (e.g. subjects reporting one medical condition were more likely to also report another medical condition), making it difficult to enter them all as independent variables. We thus collapsed all the medical conditions into a single variable of comorbidity count. We considered depression (BDI score) separately from other comorbid illnesses, as we were particularly interested in the effect of mental health. Variables that were significant at p < 0.10 were then entered as covariates in a forward step-wise general linear regression model that included the forced variables of gender and age. Variables that continued to be significant at a two-sided level of p < 0.10 were kept as main effects. Two-way interactions for the remaining variables were then assessed for significance.

All analyses were performed with SPSS version 16.0.

## Results

### Socio-demographic characteristics

Table 1 outlines the socio-demographic features of the 734 participants in the study. The prisoner population was predominantly young and male, with low education and pre-incarceration employment levels. Most had spent less than one year in prison. In the 55 subjects who were excluded because they did not complete the SF-36, there was a higher proportion of females (29.1% vs. 15.8%, p = 0.011) and Aboriginal people (41.8% vs. 28.9%, p = 0.043) than in those who did complete the SF-36.

**Table 1:** Socio-demographic characteristics of prisoner respondents (total n = 734)

Characteristic	n (percentage)
Male	618 (84.2)
Age (years)	
18 - 24	242 (33.0)
25 - 39	278 (37.9)
≥ 40	214 (29.2)
Aboriginal	212 (28.9)
Born in Australia	595 (81.1)
Time in jail (years)	
≤ 1	403 (54.9)
> 1 to ≤ 5	253 (34.5)
> 5	78 (10.6)
Marital status	
single	273 (37.2)
married/regular partner	394 (53.7)
Sexual identity	
Heterosexual	640 (87.2)
Working before entering prison	314 (42.8)
Educational status	
no formal qualification	361 (49.2)
Accommodation before entering prison	
renting	401 (54.6)
live with family/own home	255 (34.7)

### Utilities

The SF-6D and Nichol utilities for the entire sample stratified by various conditions are presented in Table 2. SF-6D utilities range from 0.620 for those reporting angina and using cardiac medication to 0.764 for those scoring none/mild symptoms on the BDI. The Nichol estimated utilities are consistently higher than the SF-6D; the average paired mean difference is 0.122 (SD 0.059, p < 0.001). The two methods, are, however, highly correlated with a Spearman correlation of 0.898 (p < 0.001).

**Table 2:** SF-6D and Nichol utilities for prisoner respondents by medical conditions.

	n (percentage)	mean SF-6D utility (SD)	mean Nichol utility (SD)
Total	734 (100)	0.725 (0.119)	0.846 (0.133)
Alcohol use			
harmful^†^	282 (38.4)	0.732 (0.115)	0.854 (0.129)
Angina/chest pain			
self-report	81 (11.0)	0.644 (0.131)	0.742 (0.161)
self-report & med*	17 (2.3)	0.620 (0.169)	0.687 (0.206)
Arthritis			
self-report	120 (16.3)	0.666 (0.116)	0.772 (0.137)
Asthma			
self-report	153 (20.8)	0.687 (0.122)	0.796 (0.142)
self-report & med*	69 (9.4)	0.656 (0.130)	0.760 (0.155)
Back problems			
self-report	211 (28.7)	0.669 (0.111)	0.778 (0.137)
Beck Depression Inventory Score			
none/minimal	418 (56.9)	0.764 (0.101)	0.898 (0.100)
moderate	153 (20.8)	0.693 (0.113)	0.813 (0.118)
severe	120 (16.3)	0.625 (0.106)	0.714 (0.132)
Cholelithiasis			
self-report	28 (3.8)	0.635 (0.140)	0.740 (0.168)
Diabetes			
self-report	25 (3.4)	0.699 (0.135)	0.804 (0.147)
self-report & med*	12 (1.6)	0.724 (0.157)	0.831 (0.175)
Epilepsy			
self-report	36 (4.9)	0.670 (0.113)	0.789 (0.148)
self-report & med*	18 (2.5)	0.647 (0.120)	0.784 (0.163)
Haemorrhoids			
self-report	66 (9.0)	0.661 (0.109)	0.770 (0.115)
Hepatitis B			
self-report	87 (11.9)	0.703 (0.119)	0.815 (0.135)
Hepatitis C			
self-report	199 (27.1)	0.704 (0.121)	0.820 (0.138)
Ab positive/viremic	178 (24.3)	0.719 (0.119)	0.839 (0.133)
correctly aware positive**	127 (17.3)	0.709 (0.120)	0.824 (0.137)
unaware positive**	51 (6.9)	0.744 (0.116)	0.879 (0.112)
correctly aware negative**	417 (56.8)	0.729 (0.118)	0.852 (0.134)
falsely believe positive**	5 (0.7)	0.809 (0.029)	0.924 (0.085)
HIV			
self-report	7 (1.0)	0.660 (0.119)	0.788 (0.144)
Hypertension			
self-report	93 (12.7)	0.672 (0.129)	0.782 (0.155)
self-report & med*	12 (1.6)	0.633 (0.176)	0.732 (0.185)
IV drug use			
used in past year	216 (29.4)	0.711 (0.119)	0.828 (0.135)
prison methadone program	92 (12.5)	0.685 (0.119)	0.792 (0.133)
Kidney condition			
self-report	11 (1.5)	0.721 (0.124)	0.793 (0.183)
Peptic ulcer			
self-report	70 (9.5)	0.665 (0.129)	0.769 (0.151)
self-report & med*	37 (5.0)	0.648 (0.124)	0.749 (0.150)
Prostate condition			
self-report	27 (3.7)	0.666 (0.105)	0.777 (0.135)
Psychiatric condition			
took psychiatric med	82 (11.2)	0.646 (0.120)	0.752 (0.152)

^†^based on WHO Alcohol Use Disorder Identification Test levels

*respondents who both self-reported the listed condition and took prescription medication for that specific illness in the past two weeks

**correctly aware positive = self-reported yes and antibody positive/viremic; unaware positive = did not self-report but antibody positive/viremic; correctly aware negative = did not self-report and antibody negative; falsely believe positive = self-reported yes but antibody negative.

In univariate analysis, prisoners had significantly lower SF-6D utilities with the following conditions than without the conditions (p ≤ 0.005 for all): angina (delta utility [Δ] = -0.090), arthritis (Δ = -0.070), asthma (Δ = -0.048), back problems (Δ = -0.078), worse BDI score (Δ = -0.071 for moderate vs. none/mild; Δ = -0.139 for severe vs. none/mild), cholelithiasis (Δ = -0.093), epilepsy (Δ = -0.057), hemorrhoids (Δ = -0.069), hypertension (Δ = -0.060), prison methadone program use (Δ = -0.043), peptic ulcer disease (Δ = -0.066), prostate condition (Δ = -0.069), and psychiatric medication use (Δ = -0.090). Self-reporters of hepatitis B had lower scores approaching statistical significance (Δ = -0.024, p = 0.074). Harmful or hazardous alcohol consumption was not associated with significantly different scores (p = 0.412). The remaining conditions that did not reach statistical significance (diabetes Δ = -0.025, human immunodeficiency virus [HIV] Δ = -0.059, and kidney condition Δ = -0.004) had 25 or fewer participants reporting the condition.

This study's original design allowed us to further explore the effect of being aware of HCV infection on HRQL. As a whole, those who were correctly aware of having active HCV infection trended towards worse SF-6D utilities than the remaining sample (Δ = -0.023, p = 0.053). However, those unaware of active infection trended towards better scores than those who were correctly aware of their hepatitis C status (Δ = 0.035, p = 0.079).

### Predictors of SF-6D utility

In univariate analysis, sociodemographic factors which correlated with worse SF-6D utilities included increasing age, female gender, increasing time spent in jail and non-heterosexual identity (p ≤ 0.01 for all). The results of a multivariate model including medical conditions are shown in Table 3. Increasing age and female gender were found to be independent predictors of lower utilities. Each additional medical illness resulted in an approximately -0.03 decrement in utility. Each increase of 1.0 in BDI score was associated with an about -0.008 utility decrease. There was a significant interaction with worse BDI score and higher comorbidity count actually slightly increasing utility. This interaction thus functions as a correction factor to adjust utility scores upwards for subjects with both poor comorbidity and BDI scores. The multivariate model was repeated using the Nichol utilities with similar results, except the interaction between BDI score and comorbidity was not significant.

**Table 3:** General linear regression model for demographic and clinical predictors of SF-6D utility in a prisoner population (n = 734)

Variable	Beta-estimate (95% confidence interval)	p value
Intercept	0.8605 (0.8335, 0.8874)	< 0.001
Age	-0.0007 (-0.0013, -0.0001)	0.023
Female	-0.0253 (-0.0462, -0.0193)	0.018
BDI score	-0.0079 (-0.0096, -0.0062)	< 0.001
comorbidity count	-0.0281 (-0.0370, -0.0193)	< 0.001
BDI score * comorbidity count	0.0009 (0.002, 0.0015)	0.009

We further explored how many prisoners with psychological needs access mental health care, and found that of subjects in our sample reporting severe BDI scores, only 43.7% were receiving counselling and only 24.2% had been taking a prescription psychiatric medication

## Discussion

Our main aim is to derive inmate-based health state utilities and our results do provide such values from a large, general prisoner population. Not surprisingly, the mean SF-6D utility (0.73) is lower than the average Australian population of 0.80 [16], as well as the average male US population aged 35 to 44 of 0.89 [17]. Such decrements of about 0.07 to 0.16 relative to the average population is a considerable amount of disutility, comparable in this study to the difference between having and not having arthritis (-0.070) or depression (-0.139). It is uncertain how much of this difference is secondary to the population's socio-demographic status, baseline number of comorbidities or effect of incarceration. The data contribute to an emerging body of literature describing the HRQL in this marginalized group. The utilities obtained may prove useful in future cost-effectiveness analyses of prisoner programs and help guide health policy.

In terms of directing health programs to issues most affecting prisoner HRQL, it is interesting that our multivariate analysis specially highlights the importance of gender and depressive symptoms. Other studies have also noted the particularly poor HRQL in female prisoners, and we agree with calls for women-oriented prison health programs [18,19]. Effective management of mental well-being is important in overall health and key to successfully returning to the community [1]. Given that participants in our sample did not frequently utilize counselling or psychiatric medications, opportunities would seem to exist to improve mental health care in jails.

The effect of psychology on HRQL is also illustrated in our specific analysis of prisoners with HCV. Similar to other research [20,21], the simple knowledge of having HCV does appear to have a negative impact on HRQL above that from the infection itself. While it may be tempting to dismiss this effect as being entirely psychological, it must be noted that knowing one is infected constitutes part of the condition. The decrement in utility is thus valid and real. Improving prisoner understanding of HCV and increasing availability to treatment options is especially important in this population with highly prevalent HCV and poor baseline mental health.

With respect to the different methods of deriving utilities, we found the Nichol utilities to be consistently higher than the SF-6D, and we are unsure as to why. Studies of SF-36 derived utilities in patients undergoing total hip arthroplasty [22] and lung transplants [23] found similar results. The Nichol utility is based on the HUI2, which, like the SF-6D, is also based on the standard gamble. Cultural differences may be at play. For example, in one study of type 2 diabetics, Euro-Qol 5D index scores based on US weights were higher than UK ones [24]. Nichol utilities are based on an American derivation as opposed to the UK SF-6D translation used in this study. From a methodological and theoretical standpoint, the respondent level SF-6D utilities are more robust than the Nichol translation. The presented utilities must be interpreted in the context of how they were derived as alternative techniques may have led to different results. The statistical significance of our findings may have changed had utilities been elicited in another manner. This study further illustrates how generic health utility instruments can range widely depending on the method of elicitation - which method to use can be controversial [3]. A more comprehensive, systematic analysis that compares the different techniques to obtain utilities from the SF-36 and other utility elicitation methods is warranted. For instance, it would be interesting to determine where the methods differ the most (e.g., low vs. high utility levels, ceiling and floor effects, responsiveness in various patient groups, etc.), although such analysis is beyond the scope of this paper.

Our study does have limitations. First, the prisoners who did not complete the SF-36 were significantly different in some socio-demographic features and thus their utilities may have also differed. However, the number of non-completers was small. We were forced to rely on self-report for diagnosing most health conditions, although when possible we also tried to corroborate this with prescription medication use. Finally, this study used data from 1996 and changes to correctional institutions since that affect health utilities may not be reflected in these scores.

## Conclusion

To the best of our knowledge, this paper provides the first utilities directly obtained from a prisoner population. The values may help provide prison-based decision and cost-effectiveness analyses with a stronger evidence base. This study highlights the importance of gender and depression on prisoner quality of life, and also how simple knowledge of HCV infection might worsen utilities. Such findings may have implications for directing prison-based health programs. Future research should include obtaining direct utilities from prisoners using standard techniques (e.g. standard gamble), replicating this study in a more current population, documenting changes in health status over time while incarcerated, exploring the HRQL impact of various prison-based health interventions and obtaining utilities from prisons in other countries.

## Abbreviations

HRQL: Health-related quality of life; NSW: New South Wales; HCV: hepatitis C virus; PCR: polymerase chain reaction; BDI: Beck Depression Inventory; SF-36: Short-Form 36; HUI2: Health Utilities Index II; US: United States; UK: United Kingdom; HIV: human immunodeficiency virus

## Competing interests

The authors declare that they have no competing interests.

## Authors' contributions

CAKYC was involved in study design, performed statistical analysis, interpreted the data and wrote the first draft. SL provided a literature review and helped draft the manuscript. GCN provided statistical support and early data interpretation, and helped draft and revise the paper. AS conceived the original idea for this paper and revised the manuscript. TB and MHL were involved in the original survey used in this paper, provided the original data and revised the manuscript. MDK was involved in data interpretation and manuscript revision. HT was involved in study design, data organization, early data interpretation and manuscript revision. All authors read and approved the final manuscript.
